# Supplementary figures and images for: Automatic reagent handling and assay processing of human biospecimens inside a transportation container for a medical disaster response against radiation
Source: PLoS One. 2022 May 20;17(5):e0268508. doi: 10.1371/journal.pone.0268508 (PMC9122182; doi:10.1371/journal.pone.0268508)

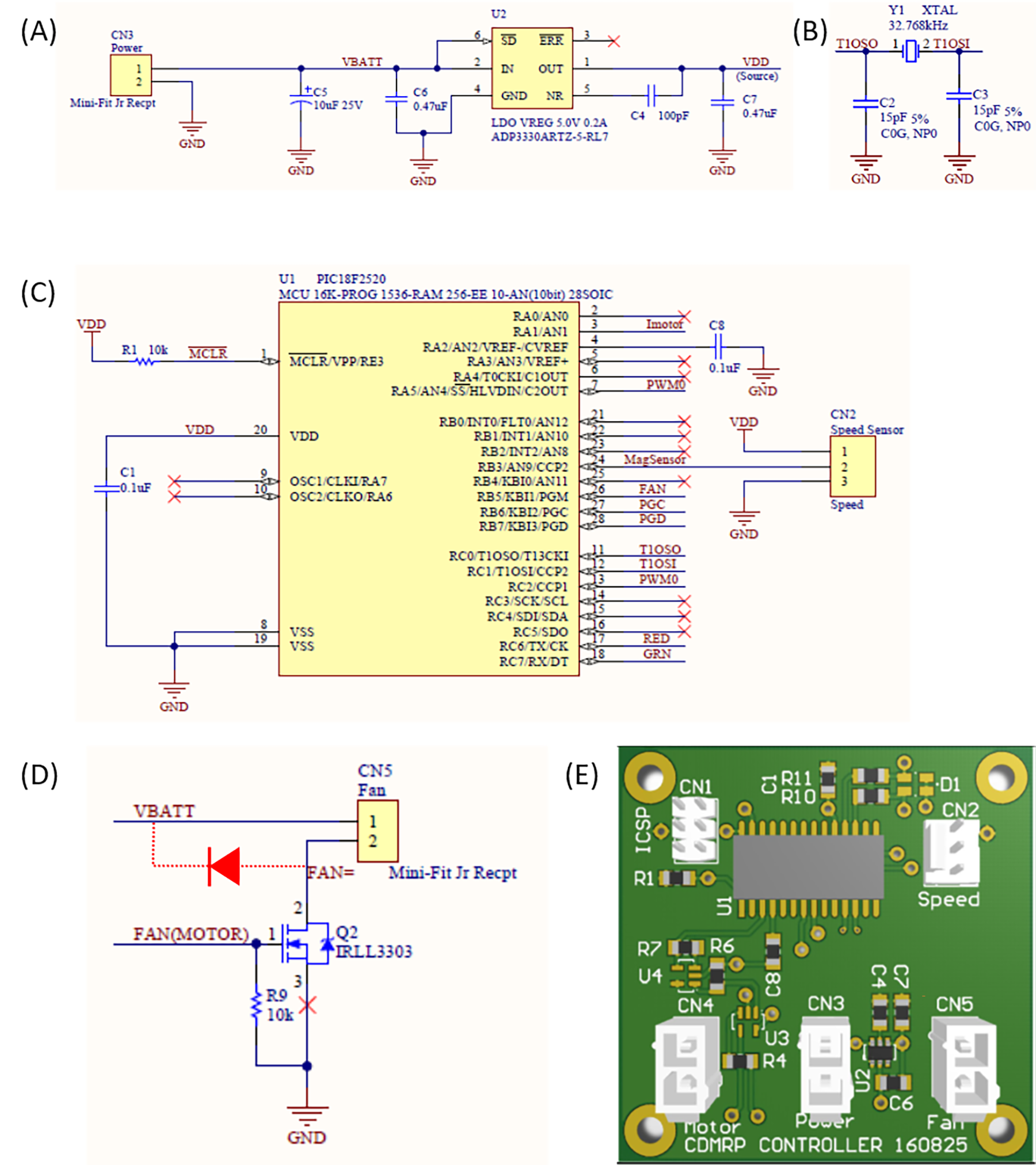

Supplement: S1 Fig — (A) a power reduction circuit from 12 to 5 V to provide power to the MCU (microcontroller unit) chip; (B) the clock circuit for timekeeping; (C) the MCU pinout and circuit diagram; (D) the fan control circuit; with an additional diode (red), the same circuit design was also used to control the motor; (E) 3D print of the PCB microcontroller (top). (TIF) [file pone.0268508.s001.tif]

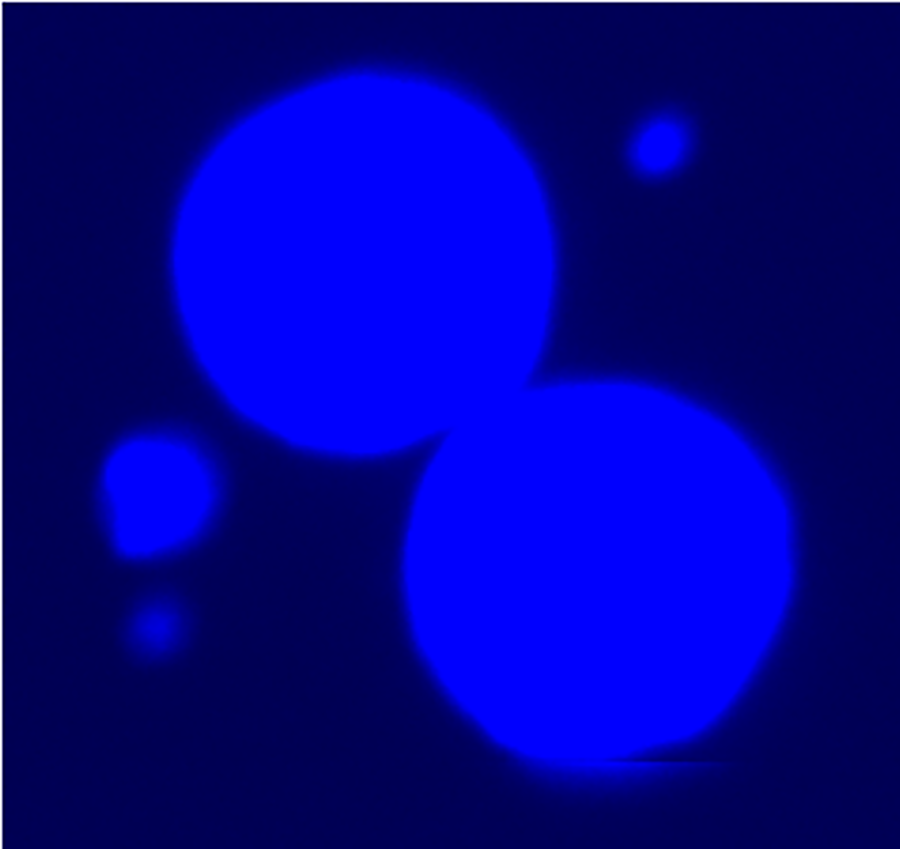

Supplement: S2 Fig — (TIF) [file pone.0268508.s002.tif]
